# Supplementary material for: Molecular Structures and Spectral Properties of Natural Indigo and Indirubin: Experimental and DFT Studies
Source: Molecules. 2019 Oct 24;24(21):3831. doi: 10.3390/molecules24213831 (PMC6865026; doi:10.3390/molecules24213831)
Supplement: Supplementary file 1 [file molecules-24-03831-s001.pdf]

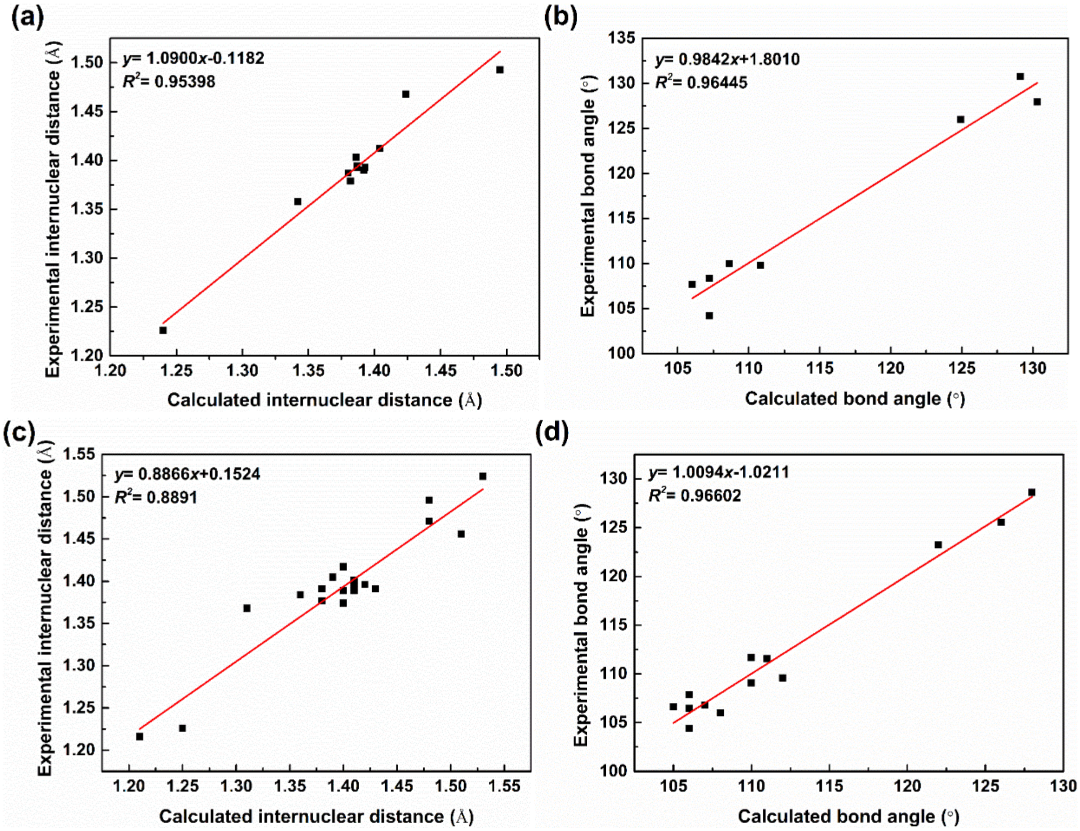

Figure S1. The correlation between computational and experimental data in literature of bond lengths and angles of indigo (a,b) and indirubin (c,d).
